# Mitochondrial dysfunction in pulmonary arterial hypertension

**DOI:** 10.3389/fphys.2022.1079989

**Published:** 2022-12-14

**Authors:** Weiwei Zhang, Bo Liu, Yazhou Wang, Hengli Zhang, Lang He, Pan Wang, Mingqing Dong

**Affiliations:** ^1^ Department of Oncology, Cancer Prevention and Treatment Institute of Chengdu, Chengdu Fifth People’s Hospital (The Second Clinical Medical College Affiliated Fifth People’s Hospital of Chengdu University of Traditional Chinese Medicine), Chengdu, China; ^2^ Department of Cardiovascular, Geratric Diseases Institute of Chengdu, Chengdu Fifth People’s Hospital (The Second Clinical Medical College Affiliated Fifth People’s Hospital of Chengdu University of Traditional Chinese Medicine), Chengdu, China; ^3^ Department of Cardiothoracic, Cancer Prevention and Treatment Institute of Chengdu, Chengdu Fifth People’s Hospital (The Second Clinical Medical College Affiliated Fifth People’s Hospital of Chengdu University of Traditional Chinese Medicine), Chengdu, China; ^4^ Department of Critical Care Medicine, The Traditional Chinese Medicine Hospital of Wenjiang District, Chengdu, China; ^5^ Center for Medicine Research and Translation, Chengdu Fifth People’s Hospital (The Second Clinical Medical College, Affiliated Fifth People’s Hospital of Chengdu University of Traditional Chinese Medicine), Chengdu, China

**Keywords:** mitochondrial dysfunction, mitochondrial metabolism, pulmonary arterial hypertension, pulmonary vasoconstriction, pulmonary vascular remodeling

## Abstract

Pulmonary arterial hypertension (PAH) is characterized by the increased pulmonary vascular resistance due to pulmonary vasoconstriction and vascular remodeling. PAH has high disability, high mortality and poor prognosis, which is becoming a more common global health issue. There is currently no drug that can permanently cure PAH patients. The pathogenesis of PAH is still not fully elucidated. However, the role of metabolic theory in the pathogenesis of PAH is becoming clearer, especially mitochondrial metabolism. With the deepening of mitochondrial researches in recent years, more and more studies have shown that the occurrence and development of PAH are closely related to mitochondrial dysfunction, including the tricarboxylic acid cycle, redox homeostasis, enhanced glycolysis, and increased reactive oxygen species production, calcium dysregulation, mitophagy, etc. This review will further elucidate the relationship between mitochondrial metabolism and pulmonary vasoconstriction and pulmonary vascular remodeling. It might be possible to explore more comprehensive and specific treatment strategies for PAH by understanding these mitochondrial metabolic mechanisms.

## Introduction

As measured by right heart catheterization, pulmonary hypertension is defined as a mean pulmonary artery pressure greater than 20 mm Hg (Hoeper et al., 2013). Furthermore, Pulmonary hypertension also can be classified as precapillary (PAWP ≤15 mm Hg) and postcapillary (PAWP >15 mm Hg) based on the pulmonary artery wedge pressure (PAWP). Experts classified pulmonary hypertension into five clinical groups based on similar pathophysiology, hemodynamic profile, clinical manifestations and treatment at the 2018 World Hypertension Symposium meeting. This includes pulmonary arterial hypertension, pulmonary hypertension that results from left-sided heart disease, pulmonary hypertension that results from lung disease or hypoxia, chronic thromboembolic pulmonary hypertension, and pulmonary hypertension with unclear or multifactorial mechanisms (Simonneau et al., 2019).

**FIGURE 1:**
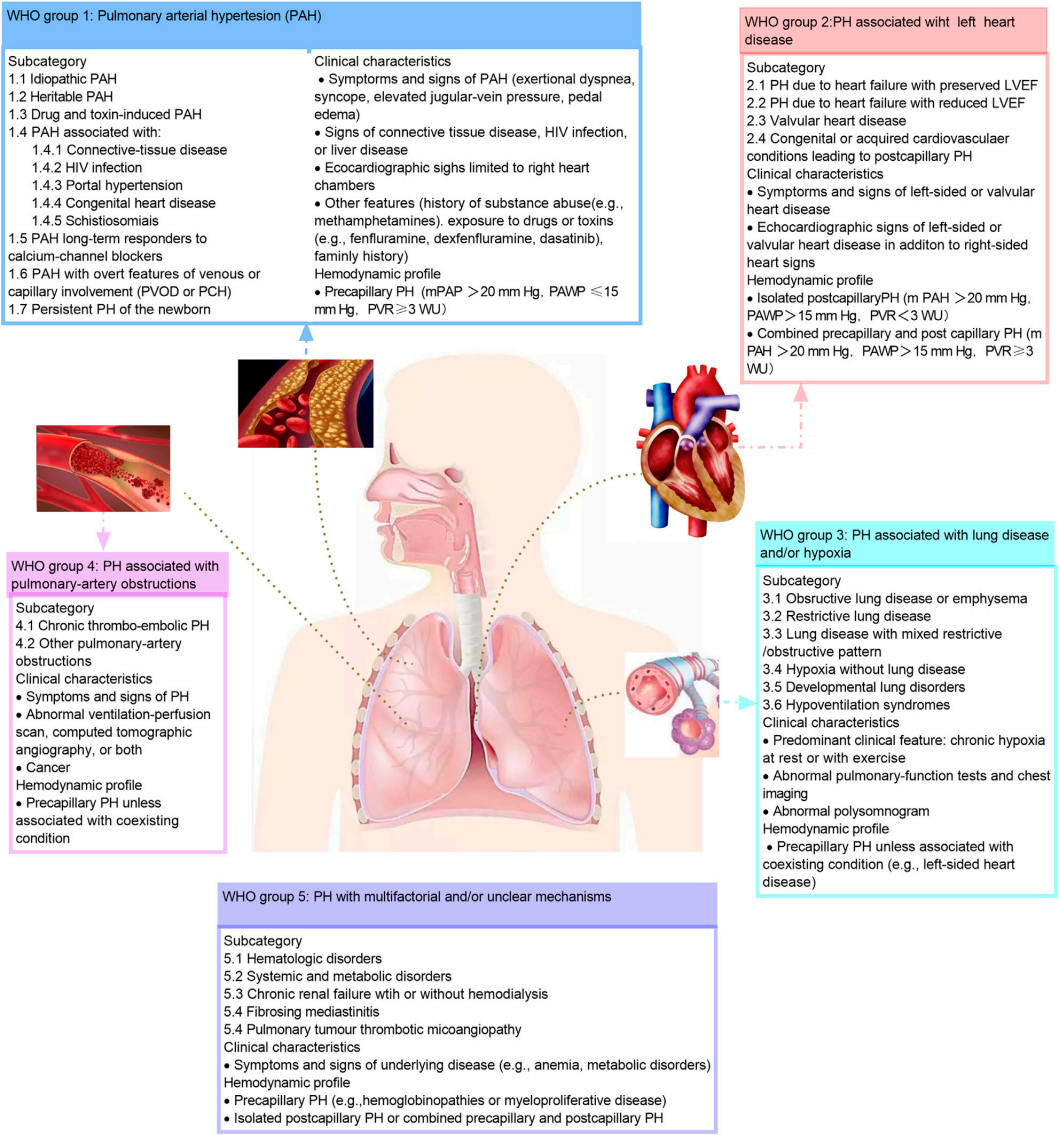
Clinical classification of pulmonary hypertension. The classification of the pulmonary hypertension based on the 2018 World Hypertension Symposium meeting. HIV, human immunodeficiency virus; LVEF, left ventricular ejection fraction; mPAP, mean pulmonary artery pressure; PAH, pulmonary artery hypertension; PAP, pulmonary artery pressure; PAWP, pulmonary artery wedge pressure; PCH, pulmonary capillary hem-angiomates; PH, pulmonary hypertension; PVOD, pulmonary arterial veno-occlusive disease; PVR, pulmonary vascular resistance; RV, right ventricular; WU, wood unites. This figure is modified from Figure 1 in Hassoun, 2021.

Pulmonary arterial hypertension (PAH) is the first clinical group of five groups of pulmonary hypertension and is the mainly content in this review. It is estimated that 2–5 patients per million people in Western countries suffer from PAH each year, and approximately 1 percent of the world’s population suffers from this disease (Humbert et al., 2006; Thenappan et al., 2018). Patients with PAH often have no specific symptoms in the early stage, and in late stage, they can present with dyspnea and fatigue. PAH is one of the difficulties in the treatment of cardiovascular disease due to its high disability, high mortality and poor prognosis (Macchia et al., 2007). The hemodynamic hallmark of PAH is precapillary pulmonary hypertension, characterized by a mean pulmonary arterial pressure greater than 20 mm Hg, a decrease in pulmonary artery wedge pressure of 15 mm Hg, and a pulmonary vascular resistance of 3 Wood units or greater based on the right heart catheterization (Simonneau et al., 2019; Humbert et al., 2022). PAH can be further divided into subgroups based on underlying etiology, such as idiopathic PAH, heritable PAH, drug- and toxin-associated PAH, etc. (McLaughlin et al., 2009; Hassoun, 2021; Ruopp and Cockrill, 2022) (Figure 1). The World Health Organization functional class is one of the strongest predictors of PAH survival (Humbert et al., 2022).

Although its pathogenesis remains unclear (Stacher et al., 2012; Tuder, 2017; Simonneau et al., 2019), it is generally accepted that PAH is a progressive and fatal vascular disease of multiple causes characterized by abnormally elevated pulmonary arterial pressure. Persistent pulmonary vasoconstriction and excessive occlusive pulmonary vascular remodeling are the main pathological changes in the formation of PAH (Chan and Loscalzo, 2008). Similar to atherosclerosis, the early pathological lesions of PAH are manifested by endothelial dysfunction and apoptosis, and the late stage manifests as hyperproliferative and anti-apoptotic endothelial and smooth muscle cells to remodel pulmonary blood vessels, which in turn increases pulmonary artery pressure and results in right ventricular dysfunction and failure (Michelakis, 2006; Xu et al., 2007; Tuder et al., 2013; Thompson and Lawrie, 2017; Thenappan et al., 2018). Mechanistically, the mechanism of pulmonary vasoconstriction and remodeling is very complex, including abnormal activation of growth factor signaling pathway (Dumas et al., 2018; Ma et al., 2019), abnormal ion channel function (Lambert et al., 2019), inflammatory injury (Siques et al., 2021), oxidative stress (Siques et al., 2021), abnormal energy metabolism (Harvey and Chan, 2017), oxygen glycolysis (Liang et al., 2022), fatty acid oxidation (Harvey and Chan, 2017), etc. Mitochondria are recognized sensors of oxygen and fuel, and the most important energy-producing site of the body (Dromparis and Michelakis, 2013). However, mitochondria are central to the pathogenesis of PAH, which also represents all the functions of mitochondria to date. Mitochondria may be the cornerstone of new therapeutic approaches (Dromparis et al., 2010). Mitochondrial dysfunction not only causes energy metabolism disorders in pulmonary artery smooth muscle cells (PASMCs), but also produces a large number of reactive oxygen species (ROS), increases oxidative stress and activates inflammatory responses (Culley and Chan, 2018).

The treatments for PAH are extremely limited. Currently, the main drugs can be used to prolong life and improve quality of life in patients with PAH, including the enhancer of biological pathway of nitric oxide-cyclic guanosine monophosphate, prostacyclin pathway agonists, and endothelin pathway antagonists (Ruopp and Cockrill, 2022). However, these treatments are not specific, neither reversed PAH nor significantly extended lifespan (Macchia et al., 2007; McLaughlin et al., 2009). While reversing pulmonary vasoconstriction, these drugs also cause great damage to systemic blood vessels (such as renal blood vessels, cerebral blood vessels, etc.). This may be related to the complex pathogenesis of PAH. Excitingly, considering the difference between pulmonary circulation and systemic circulation, many researchers have begun to study targeted drugs that specifically recognize pulmonary blood vessels to reverse pulmonary vascular remodeling. The purpose of this paper is to review mitochondrial metabolic pathways and related mechanisms and summarize current state-of-the-art treatments for PAH.

## Mitochondria and pulmonary vascular remodeling

Persistent proliferation and resistance to apoptosis of pulmonary vessels are the main causes of pulmonary vascular remodeling (Veyssier-Belot and Cacoub, 1999). The mechanism of pulmonary vascular remodeling is complex. It involves the damage of bone morphogenetic protein receptor 2 signaling pathway (Hautefort et al., 2019), abnormal activation of growth factor signaling pathway (Dumas et al., 2018; Ma et al., 2019), abnormal ion channel function (such as KCNK3) (Lambert et al., 2019), inflammatory damage (Siques et al., 2021), oxidative stress (Siques et al., 2021), abnormal energy metabolism (Harvey and Chan, 2017), etc. Mitochondria are the most important energy production sites in the body. Mitochondrial dysfunction is well known to be closely associated with PAH development and occurrence. Abnormal mitochondrial function not only causes energy metabolism disorders in PASMCs, but also produces a large number of ROS, increases oxidative stress and activates inflammatory responses (Culley and Chan, 2018). Therefore, we will elucidate the relationship between mitochondrial dysfunction and PAH from aspects of mitochondrial quality control, DNA damage, metabolic dynamics, autophagy, and ROS production.

### Mitochondrial quality control

**FIGURE 2:**
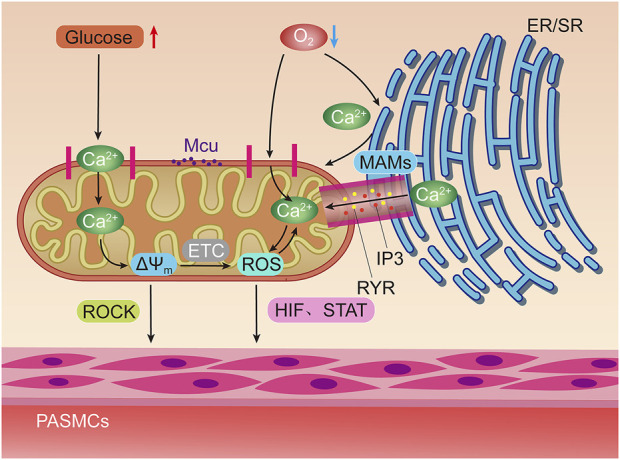
Hypoxia and elevated glucose can stimulate mitochondria to induce pulmonary artery smooth muscle contraction through different ways. Hypoxia can directly stimulate mitochondrial Ca^2+^ uniporter (MCU) to increase Ca^2+^ uptake, or activate inositol triphosphate receptors (IP3R) and ryanodine receptors (RyR) on Mitochondrial associated endothelial reticulum (MAMs) to increase the concentration of oxygen free radicals (ROS) in mitochondria by releasing high concentrations of Ca^2+^ into the mitochondrial matrix. Positive feedback regulation can be formed between ROS and Ca^2+^. The increase of ROS can promote the contraction of pulmonary artery smooth muscle through hypoxia inducible factors (HIF) and signal transducer and activator of transfer (STAT). Glucose oxidation can increase mitochondrial membrane potential (△Ψm) Concentration, thereby activating Rho/Rho kinase signal and other mechanisms to induce pulmonary artery smooth muscle contraction.

The mitochondria are highly dynamic, maternally inherited organelles that provide energy for the cell through oxidative phosphorylation, integrating various metabolic pathways, regulating apoptosis, and maintaining calcium homeostasis. As the center of energy metabolism, mitochondrial defects will lead to a variety of complex human diseases. However, human mitochondria will maintain their own integrity and homeostasis through quality control (Nunnari and Suomalainen, 2012). Mitochondrial quality control will ensure cellular homeostasis by coordinating pathways such as protein homeostasis, biogenesis, mitochondrial dynamics, and mitophagy *in vivo* (Picca et al., 2018) (Figure 2).

#### Mitochondrial protein balance

As one of the important organelles for protein production and secretion *in vivo*, changes in endoplasmic reticulum homeostasis can lead to intracellular unfolded protein responses, resulting in mitochondrial protein imbalance. Studies have shown that mitochondrial protein imbalances can lead to metabolic disease, inflammation, neurodegenerative diseases and cancer (Wang and Kaufman, 2012). However, the relationship between mitochondrial protein imbalance and PAH is currently unclear. But James and others have suggested that mitochondrial protein homeostasis may play an important role in the pulmonary circulation (James et al., 2020). In this study, the authors found that altered mitochondrial proteostasis promotes glycolysis and initiates metabolic reprogramming by reducing protein clearance and detoxification, resulting in sustained constriction and proliferation of pulmonary vasculature (James et al., 2020).

#### Mitochondrial biogenesis

During mitochondrial biogenesis, mitochondrial DNA (mtDNA) is synthesized under the control of peroxisome proliferator-activated receptor-γ coactivator-1α (PGC-1α). In this process, the mitochondrial transcription factor a (TFAM) nuclear gene is the final effector of mtDNA transcription and replication. PGC-1α can increase the expression of TFAM by stimulating nuclear respiratory factor-1 (NRF-1), nuclear respiratory factor-2 (NRF-2) and estrogen-related receptor-α (ERR-α) (Jornayvaz and Shulman, 2010; Popov, 2020). Hypoxia, loss of peroxisome proliferator-activated receptor-γ (PPARγ), or decreased PGC1α reduced mitochondrial mass by increasing mitochondrial hydrogen peroxide production and mitochondrial fission, thereby impairing mitochondrial biological function and promoting PASMCs proliferation (Yeligar et al., 2018).

As a nicotinamide adenine dinucleotide-dependent deacetylase, Sirtuin 1 (SIRT1) can enhance mitochondrial biogenesis by deacetylating and activating PGC-1α. One study found that SIRT1 activators inhibited the proliferation of mouse and human PASMCs under hypoxia-induced conditions, and SIRT1 knockout mice exhibited higher vascular remodeling than non-knockout mice. This effect is related to the activation of mitochondria caused by the overexpression of mitochondrial markers and the down-regulation of PGC-1a (Zurlo et al., 2018). Additionally, the vasodilator nitric oxide (NO) controls cellular respiration and mitochondrial biogenesis (Afolayan et al., 2016). In vascular smooth muscle cells, NO is an effective vasodilator. It promotes PASMCs and vasodilation by activating soluble guanylate cyclase (sGC) to increase the production of cGMP, thereby reducing calcium influx (Furchgott and Zawadzki, 1980). In a fetal sheep model of persistent PAH, inhaled NO can reduce mitochondrial DNA copy number, electron transport chain complex subunit and adenosine triphosphate levels to promote mitochondrial biogenesis, and improve mitochondrial dysfunction (Afolayan et al., 2016).

Nitric oxide synthase isoenzymes include neuronal NOS, inducible NOS and endothelial NOS (eNOS) (Coggins and Bloch, 2007). Among them, eNOS mainly comes from pulmonary vascular endothelium and is also the main source of NO in pulmonary circulation. Many studies suggest that the production of NO in PAH is significantly reduced. Although many studies have found that the production of NO in PAH patients is impaired, their results are contradictory (Coggins and Bloch, 2007; Lázár et al., 2020). The reason is that some studies found that chronic hypoxia can reduce the expression of eNOS in pulmonary vessels (Ziesche et al., 1996; Ostergaard et al., 2007), while other studies found that chronic hypoxia can induce the expression of all NOS subtypes, including eNOS (Fagan et al., 2001; Ward et al., 2005). Lázár et al. (2020) study have shown that the decreased expression or dysfunction of endothelial nitric oxide synthase (eNOS) can eliminate the NO signal transduction in PAH. It can be seen that targeted inhibition of eNOS degradation may repair mitochondrial function, thereby reducing the occurrence and development of PAH. Another study also has shown that miR-1226-3p can target Profilin-1 to increase the content of eNOS and NO in PAH rats, thereby protecting rats from PAH induced injury (Jian and Xia, 2021). It can be seen that the role of eNOS in PAH is controversial at present, and more research is needed to confirm it.

#### Mitochondrial dynamics

The phenomenon of continuous division and fusion of mitochondria is called mitochondrial dynamics. Even under metabolic and environmental pressures, mitochondrial fusion can still transfer gene products between mitochondria to achieve optimal function. Mitochondrial division is crucial to maintain the number and proper distribution of mitochondria in daughter cells (Adebayo et al., 2021). When this balance is disturbed, it may cause many diseases, including cancer, PAH, etc.

##### Mitochondrial fission

Mitochondrial fission mainly depends on the dynamin-related protein 1 (DRP1) in the cytoplasm and the proteins on the outer membrane of mitochondria (OMM), such as mitochondrial fission 1 (FIS1), mitochondrial fission factor (MFF), mitochondrial dynamics protein of 49 (MID49) and mitochondrial dynamics protein of 51 (MID51) (Sabouny and Shutt, 2020). DRP1 inhibitor can inhibit the development of PAH by reversing mitochondrial function (Parra et al., 2017; Tian et al., 2018; Zhuan et al., 2020; Wu et al., 2021; Xiao et al., 2022). Extracellular regulated kinase, p38 Mitogen-Activated protein kinases (MAPK), camp-dependent protein Kinase, adenosine monophosphate-activated protein kinase and sirtuin can phosphorylate DRP1 (Ren et al., 2020). The phosphorylated DRP1 translocates to the OMM and binds with FIS1, MID49, MID 51, MFF, and other receptors to form a circular structure, thus dividing mitochondria. Among them, the contraction of mitochondrial inner chamber is considered as the starting event of effective mitochondrial division (Loson et al., 2013). In this process, phosphorylation of serine 637 and 656 sites can inhibit mitochondrial division by reducing DRP1 activity (Chang and Blackstone, 2007; Cribbs and Strack, 2007), while phosphorylation of serine 616, 579, and 600 sites can promote mitochondrial division by enhancing DRP1 activity (Wikstrom et al., 2014; Kashatus et al., 2015; Prieto et al., 2016; Fu et al., 2017; Ko et al., 2017; Li et al., 2019).

In PAH, DRP1 is a key regulator of PASMCs proliferation. Many studies have shown that down-regulation of DRP1 can reverse the formation of PAH. The expression of DRP1 in PASMCs of PAH patients was significantly increased, and the proliferation of PASMCs was induced by chemical activation of hypoxia-inducible factor-1α (HIF-1α) to induce DRP1 Ser616 phosphorylation (Marsboom et al., 2012). Under hypoxia, HIF-1α is a key factor for mitochondrial dysfunction and inducing PASMCs proliferation and apoptosis (Chen et al., 2019). HIF-1α can regulates the mitochondrial dynamics of hypoxic pulmonary vascular remodeling by directly regulating the expression of DRP1. Mitochondrial division inhibitor 1 (Mdivi-1) can inhibit mitochondrial division by binding to allosteric sites on DRP1, and further studies have found that the anti-proliferation effect of Mdivi-1 is attributed to the induction of cell cycle arrest in G2/M phase (Marsboom et al., 2012). In addition to inhibiting mitochondrial division, Mdivi-1 also inhibits glycolytic metabolism in PASMCs (Parra et al., 2017). However, it is interesting that some studies have found that mdivi-1 is a reversible inhibitor of mitochondrial complex I, rather than a specific DRP1 inhibitor, which can rapidly inhibit complex I dependence rather than complex II dependence. Therefore, it may be necessary to further explore the molecular mechanism between DRP1 and PAH, so as to find more specific molecular targeted drugs (Bordt et al., 2017).

FIS1, MFF, MID49, and MID51 on the OMM also have some effect on mitochondrial division. FIS1, MFF, MID49, and MID51 could recruit DRP1 to the mitochondrial outer membrane and promote mitochondrial division (Loson et al., 2013). However, it should be noted that MIDs are also able to promote mitochondrial fission in the absence of FIS1 and MFF (Chen K. H. et al., 2018). Furthermore, the increased expression of MIDs in PASMCs can promote DRP-1 mediated mitochondrial division, and promotes PAMCs proliferation and reduce their apoptosis (Chen K. H. et al., 2018).

#### Mitochondrial fusion and PAH

Mitochondria promote mitochondrial fusion through optical atrophy 1 (OPA1) and mitofusin 1 (MFN1) and mitofusin 2 (MFN2) between the outer and inner membranes of adjacent mitochondria. MFN1 and MFN2 mediate mitochondrial outer membrane fusion, while OPA1 mediates mitochondrial inner membrane fusion (Sabouny and Shutt, 2020). Inhibition of MFN1, MFN2 or OPA1 will reduce mitochondrial fusion and promote mitochondrial fission. Therefore, MFN1, MFN2 or OPA1 may also be targets for intervention in PAH.

Increased MFN2 expression can increase mitochondrial fusion, reduce mitochondrial fission, reduce PASMCs proliferation, and increase PASMCs apoptosis (Lu et al., 2016; Zhu et al., 2017). Ryan’ study (Ryan et al., 2013) found that decreased mitochondrial fusion is also a complementary means of mitochondrial division, and the study identified MFN2 down regulation as a proliferation pathway. In human PAH and two established rodent PAH models, MFN2 deficiency led to excessive proliferation of PASMCs. The basis of MFN2 downregulation is related to the down-regulation of PGC1a, a transcriptional coactivator of MFN2. Reduced expression of MFN2 and PGC1a contributes to mitochondrial.

MFN1 is involved in the proliferation of PASMCs in patients with PAH (Omura et al., 2019). Ma et al. (2017) showed that hypoxia upregulated the expression of MFN1 in PASMCs both *in vivo* and *in vitro*, and that MFN1-mediated mitochondrial homeostasis and PASMCs proliferation were regulated by miR-125α. This also provides a theoretical basis for the treatment of PAH through miR-125α-MFN1 pathway. Recently, researchers found that O-[3-piperidino-2-hydroxy-1-propyl]-nicotinic amidoxim (BGP-15) promotes mitochondrial fusion by promoting the GTPase activity and self-aggregation of OPA1, but this role has not been confirmed in PAH (Szabo et al., 2018).

#### Mitochondrial mitophagy

Mitochondrial health is primarily maintained through mitochondrial dynamics and mitophagy. Mitophagy plays an important role in the quality control of mitochondria by eliminating damaged mitochondria. Fission can also isolate the damaged and irreparable mitochondria and promote mitophagy. Therefore, mitochondrial dynamics and mitophagy are closely related. Mitophagy is a form of selective autophagy. When mitochondrial damage occurs, the translocation mediated by TIM23 complex is inhibited, resulting in the retention of the transmembrane domain of PTEN-induced putative kinase 1(PINK1), its integration into the outer membrane, escape from degradation, autophosphorylation, and phosphorylation. This leads to the accumulation and activation of E3 ubiquitin-protein ligase parkin (Parkin) (Hoshino et al., 2019), which promotes the ubiquitination of mitochondrial outer membrane proteins (Ordureau et al., 2014). This ubiquitination of mitochondrial outer membrane proteins leads to the participation of autophagy receptors to activate mitophagy. Autophagy receptors include CALCOCO2 (also known as NDP52), Optineurin (OPTN), Tax1-binding protein (TAX1BP1), and FUNDC1 (Lazarou et al., 2015; Liu et al., 2022). FUNDC1 belongs to the mitophagy receptor. Study Liu et al. (2022) found that the overexpression of FUNDC1 can promote the mitochondrial phagocytosis and cell proliferation of PASMCs by activating ROS-HIFA pathway, thus leading to pulmonary vascular remodeling. Study Ma et al. (2022) found that apoptosis-inducing factor (AIF) deficiency in hypoxia can lead to mitochondrial complex I instability, resulting in oxidative phosphorylation (OXPHOS) disorder, high levels of mitochondrial ROS production, and increased expression of Pink and Parkin, leading to excessive mitophagy.

However, some studies have found that UCP2 is also involved in mitochondrial autophagy. As a negative ion transport protein, uncoupling protein 2 (UCP2) is mainly located in the inner membrane of mitochondria. Loss of UCP2 will lead to the reduction of Ca^2+^ from the endoplasmic reticulum into mitochondria in PASMCs, leading to the reduction of mitochondrial Ca^2+^, and then inhibit mitochondrial function. A prospective study found that UCP2 was positively correlated with the severity of PAH. Furthermore, the mean pulmonary artery pressure and vascular remodeling in Sirt3 and Ucp2 double knockout PAH mice were significantly increased, and the mitochondrial function in pulmonary arteries was significantly inhibited. UCP2 of pulmonary vascular endothelial cells also can induce mitochondrial phagocytosis, insufficient mitochondrial biosynthesis and increased endothelial cell apoptosis through PTEN and kinase 1, thus leading to pulmonary vascular remodeling (Haslip et al., 2015).

### Mitochondrial DNA damage

When continuously exposed to cellular metabolites and exogenous factors, the structural integrity of mitochondrial DNA may be destroyed. It can range from a simple structural change to a complex one. Depending on the type of DNA damage, corresponding DNA damage repair pathways are activated, aiming to restore DNA double-strands, prevent damaged DNA replication, and ensure intact healthy DNA transmission. And initiates apoptotic signals when the damage cannot be repaired, leading to cell death (Sharma and Aldred, 2020). The dysregulated DNA-damage response (DDR) pathway is one of the important causes of nuclear DNA damage (Ranchoux et al., 2016). Sharma’s review also showed that the detection DDR genes involved in the pathogenesis of PAH include Poly [ADP-ribose] polymerase 1 (PARP-1) and Proto-oncogene Serine/Threonine Kinase (PIM-1), Eyes Absent Homolog 3 (EYA3) and checkpoint kinase-1(CHK1), when these genes are up-regulated in PASMCs of PAH, they lead to increased DNA repair and proliferation and decreased apoptosis, thereby promoting PASMCs proliferation. Oxidative stress and inflammation are another important cause of nuclear and mitochondrial DNA damage (Cline, 2012).

### ROS and mitochondria

Mitochondria is the important organ of intracellular ROS. PASMCs exposed to hypoxia will produce a large amount of ROS, especially mitochondria-derived ROS. Although many studies found that ROS abnormal expression is common in PAH patients (Archer et al., 2008; Aggarwal et al., 2010; Aggarwal et al., 2013). But interestingly, Biochem’s study found that although the ROS level in PASMCs was increased, the ROS level in mitochondria was actually decreased (Jin et al., 2016). This may be related to NADPH oxidase system. Zhang et al. (2016) found that DRP1 has a positive feedback effect on ROS. The interaction between them leads to pulmonary vascular remodeling by promoting mitochondrial fission and inhibiting apoptosis of PASMCs under hypoxia. Umezu et al. (2020) found that inhibiting the expression of DRP1 could significantly reduce the concentration and activity of ROS in mitochondria of macrophages, thereby inhibiting the growth and migration of vascular smooth muscle cells. It is suggested that DRP1 modulation promotes the intimal thickening of injured blood vessels by promoting macrophage mediated inflammatory response, and DRP1 modulation in macrophages may be a potential therapeutic target for vascular diseases.

It is also found that FUNDC1 is closely related to ROS generation, because FUNDC1 can increase ROS generation and HIF1-α to induce PASMCs proliferation (Liu et al., 2022). In addition, hypoxia can cause a significant increase in FUNDC1. Studies by Ma et al. (2022) found that *in vitro*, the mitochondrial complex I would lead to the loss of the activity of the apoptosis inducing factor under the induction of hypoxia, leading to the damage of oxidative phosphorylation, increased glycolysis and increased ROS release. DRP1, FUNDC1, and AIF are related to ROS, and these factors may become new targets for the treatment of PAH.

## Mitochondria and pulmonary vasoconstriction

**FIGURE 3:**
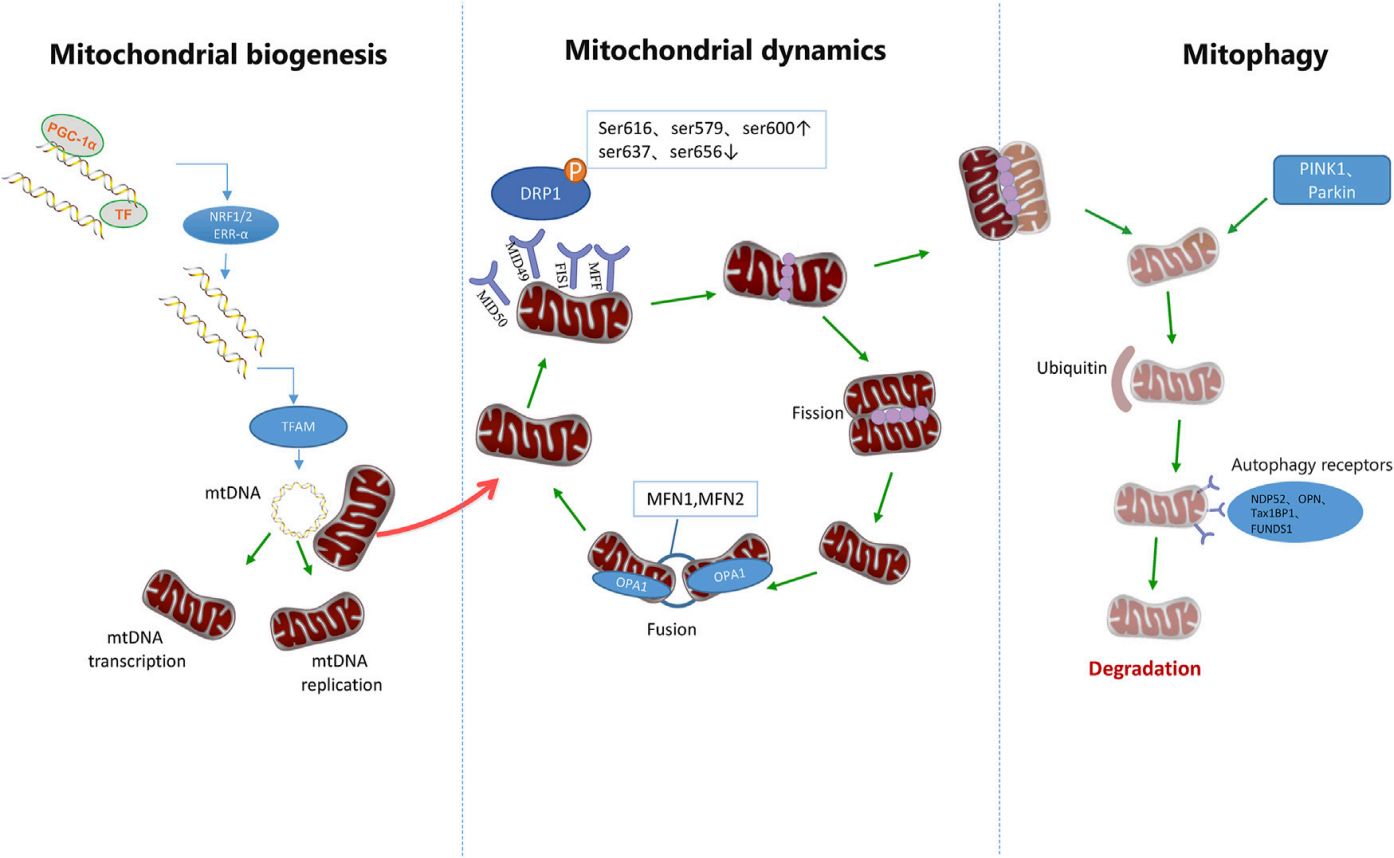
Mitochondrial quality control pathways. Mitochondrial homeostasis is ensured through the coordination of mitochondrial biogenesis, dynamics, and mitophagy. The biogenetic pathway of mitochondria is initiated by PGC-1 and then stimulates the nuclear transcription factors NRF-1, NRF-2, and ERR-α. It is then initiated by increasing the expression of TFAM, which is the final effector of mtDNA transcription and replication. After translocation of phosphorylated DRP1 to mitochondrial OMM, it combines with Fis1, MID49/51, and MFF receptors to form a ring structure. Further contraction of this structure can promote mitochondrial division. However, DRP1 phosphorylation at Ser637 and Ser656 inhibits mitochondrial division, and DRP2 phosphorylation at Ser616, Ser579, and Ser600 promotes mitochondrial division. Mitochondrial fusion is accomplished by the fusion of the outer and inner membranes of adjacent mitochondria. MFN1 and MFN2 mediate mitochondrial outer membrane fusion, while OPA1 mediates mitochondrial inner membrane fusion. Mitochondrial components eventually circulate through a special autophagic pathway, known as mitochondrial phagocytosis. PINK1 and Parkin can activate mitosis of mitochondria and promote ubiquitination of outer membrane proteins of mitochondria, thus further promoting autophagic receptors such as NDP52, OPTN, TAX1BP1, and FUNDS1 to participate in the activation of mitosis.

Pulmonary artery vasoconstriction is another factor of PAH. More and more evidences indicate that the dysfunction of mitochondria is closely related to pulmonary artery vasoconstriction. The following will describe the changes of mitochondrial functions after hypoxia and the mechanism of correlation between the dysfunction of granular bodies and pulmonary artery contraction when glucose metabolism is abnormal (Figure 3).

### Hypoxia and pulmonary artery constriction mechanism

Pulmonary vasoconstriction and remodeling are two main pathological features of hypoxic pulmonary hypertension. Pulmonary vasoconstriction can be divided into acute phase and persistent hypoxic pulmonary vasoconstriction according to the time of exposure to hypoxia. The acute phase hypoxic pulmonary vasoconstriction is characterized by rapid pulmonary vasoconstriction reaction caused by hypoxia, which means that when acute hypoxia causes the alveolar partial oxygen pressure to drop to a certain threshold, the pulmonary vessels rapidly produce reversible contractile reaction. Persistent hypoxic pulmonary vasoconstriction refers to the irreversible hypoxic pulmonary vascular remodeling caused by long-term alveolar hypoxia (Zhao Enqi and Ye 2021). This section mainly describes the main mechanisms of mitochondrial dysfunction induced by hypoxia.

#### Calcium ion channel and pulmonary vasoconstriction

It is well known that under hypoxia, the function of mitochondrial ion channels in PASMCs will be significantly affected, resulting in impairment of various functions including mitochondrial oxidative phosphorylation and ATP synthesis efficiency, regulation of signal transduction and enzyme activity in cells and cell matrix, as well as cell proliferation and apoptosis (Wang X. et al., 2021). Mitochondria are composed of the inner and outer mitochondrial membranes and the mitochondrial matrix and membrane gap (Mukherjee et al., 2021). Among them, the mitochondrial inner membrane has high ion selectivity, and its permeability plays an important role in maintaining the ion concentration inside and outside the mitochondria. The selectivity of the outer membrane of the mitochondria is low, allowing free ingress and egress of ions (Singh, 2021). The abnormal ion concentration gradient on the inner and outer membrane of mitochondria is significantly related to the occurrence and development of early pulmonary artery contraction, late pulmonary artery remodeling, myocardial ischemia-reperfusion injury and other diseases (Wang X. et al., 2021).

Mitochondrial ion channels include calcium (Ca^2+^), potassium (K^+^), sodium (Na^+^) and some anion channels. Anion channels can participate in the occurrence and development of cardiovascular diseases by maintaining the energy and material metabolism balance of mitochondria and cells, and regulating the calcium cycle of mitochondria. Mitochondrial K^+^ channels have two biological functions. One is to regulate the K^+^ concentration in mitochondria to maintain the stability of mitochondrial volume, promote the expression of enzymes related to mitochondrial energy metabolism, and protect cells. On the other hand, it participates in the oxidation and phosphorylation of mitochondria (Moudgil et al., 2006). The uptake of K^+^ through this channel can partially compensate for the charge change caused by the proton pump transport of H^+^, thus maintaining the stability of mitochondrial transmembrane potential. K^+^ channels may also participate in pulmonary vascular tone and remodeling. Despite all this, Calcium channel is the most important channel. Calcium channels are the most accurate and mature (Alevriadou et al., 2017).

The contraction of PASMCs caused by hypoxia is the key factor to trigger hypoxic pulmonary vasoconstriction, and the increase of intracellular Ca^2+^ level is one of the main reasons for the contraction of PASMCs (Jain et al., 2020). Hypoxia can induce the release of Ca^2+^ from intracellular calcium stores, promote the uptake of Ca^2+^ by mitochondria, and lead to the increase of the concentration of Ca^2+^ in cells, thus triggering hypoxic pulmonary vasoconstriction. Mitochondria in PASMCs mainly act as sensors in physiological hypoxia (Zhao Enqi and Ye 2021).

Ca^2+^ uptake by mitochondrial must pass through the outer and inner membrane of mitochondria. Hydrogen ions move through the mitochondrial inner membrane through the electron transport chain to form a concentration gradient. It accumulates electrochemical driving force for calcium ion transport to mitochondria. Mitochondrial Ca^2+^ uniporter (MCU) is the main pathway of mitochondrial Ca^2+^ uptake, which exists in the inner membrane of mitochondria. Mitochondrial Ca^2+^ uptake-1 (MICU1) is a part of MCU complex, and only Ca^2+^ is allowed to enter mitochondria. Ca^2+^ first passes through the outer membrane of mitochondria through voltage dependent anion channel 1, and then passes through the inner membrane of mitochondria through MCU to enter mitochondria (Klapper-Goldstein et al., 2020). MCU is highly selective to Ca^2+^, which is the main way for mitochondria to absorb Ca^2+^ (Baradaran et al., 2018). When the concentration of Ca^2+^ in the cytoplasm is low, MCU1 can prevent mitochondria from taking Ca^2+^ by setting a threshold, thus playing the role of gatekeeper (Payne et al., 2017).

Studies have shown that mitochondrial membrane potential, as a driving force, promotes the transfer of Ca^2+^ to mitochondria (Woods et al., 2019). MCU mediated mitochondrial uptake of Ca^2+^ plays an important role in signal transduction, bioenergetics, and cell contraction, proliferation, and apoptosis. Its imbalance is related to a variety of human diseases. Hypoxia can lead to the release of Ca^2+^ from intracellular calcium stores, and lead to the influx of extracellular Ca^2+^ by stimulating calcium channels on the cell membrane, thus leading to intracellular Ca^2+^ overload. MCU can remove excessive Ca^2+^ in the cytoplasm, and mitochondria also use these ions to generate cell energy (Baughman et al., 2011). Persistent hypoxia can damage the function of MCU, reduce the concentration of ROS in mitochondria, and then promote the contraction and proliferation of PASMCs.

Mitochondrial associated endoplasmic reticulum (MAMs) is the physical basis of communication between endoplasmic reticulum (ER) and mitochondria (Ahsan et al., 2021). The transfer of Ca^2+^ from ER to mitochondria depends on the specific calcium channels on the MAMs surface. The site where calcium stores release Ca^2+^ forms high calcium cytoplasm at MAMs and reaches a high concentration of Ca^2+^ in a short time, which is much higher than the concentration of Ca^2+^ in cytoplasm. Ca^2+^ in ER and sarcoplasmic reticulum (SR) can be released into mitochondrial matrix through inositol triphosphate receptors (IP3R) or ryanodine receptors (RyR) on their surfaces. Endoplasmic reticulum mitochondria interaction occurs in the whole network, and Ca^2+^ is one of the most important signals used by organelles for communication (Zhao Enqi and Ye 2021). The Ca^2+^ released by SR is the key to excitation contraction coupling (Sydykov et al., 2021).

The main site of ROS production induced by hypoxia is respiratory chain complex III. Recurrent inhibition of mitochondrial complex III induces chronic pulmonary vasoconstriction (Rafikova et al., 2018). Hypoxia can induce the production of ROS in mitochondria and increase the concentration of Ca^2+^ in PASMCs, thereby promoting the uptake of Ca^2+^ by mitochondria in PASMCs, which may play an important role in hypoxic pulmonary vasoconstriction and its related PAH (Yang et al., 2020). Studies have shown that the Riske Fe/S protein in complex III can mediate the production of ROS during hypoxia, thereby increasing the level of Ca^2+^ in PASMCs (Truong et al., 2019). RU360, an inhibitor of mitochondrial Ca^2+^ uptake, inhibits mitochondrial Ca^2+^ uptake by inhibiting the production of ROS induced by hypoxia in mitochondria, and finally inhibits hypoxia induced mitochondrial Ca^2+^ overload. Under hypoxia, Riske Fe/S protein dependent mitochondrial ROS production in respiratory chain complex III can activate phospholipase C-γ 1 causes IP3 generation, IP3R channel opens and releases Ca^2+^. In addition, the levels of Endothelin-1 (ET-1) and ET-A in PAH patients were increased. ET-A stimulates calcium release into the cytoplasm by increasing the concentration of IP3, leading to PASMCs contraction. ET-B, on the other hand, reduces the concentration of IP3 to cause vasodilation and clearance of ET-1 (Chaumais et al., 2015). Therefore [ROS] mito plays an important role in hypoxia induced Ca^2+^ release of SR and contraction of PASMCs. The increase of [Ca^2+^] mito will produce a large amount of ROS, which will lead to the opening of mitochondrial permeability transition pore, and finally lead to the increase of [Ca^2+^]i, forming a positive feedback mechanism. At the same time, ROS and redox couple in PASMCs can also inhibit oxygen sensitive voltage gated potassium channel Kv, activate voltage gated calcium channel, and then increase [Ca^2+^]i, leading to PASMCs contraction and hypoxic pulmonary vasoconstriction.

#### Mitochondrial ROS and pulmonary vasoconstriction

It has been shown that hypoxic stimulation of pulmonary vasoconstriction is due to inhibition of mitochondrial function as cellular oxygen receptors (Michelakis et al., 2004). The intracellular ROS include free radical superoxide (O_2_-), hydroxyl radicals (OH-), non-free radical hydrogen peroxide (H_2_O_2_), and oxygen negative ions (O-), which are mainly generated from mitochondria, reduced nicotinamide adenine dinucleotide phosphate (NADPH) oxidase, xanthine oxygenase and nitric oxide synthase (Khan et al., 2021).

Mitochondrial respiratory chain is the main source of biological ROS (Skulachev, 2007). The active oxygen produced mainly refers to the single electron reduction products of oxygen, O_2_- and its derived HO_2_-, OH-, H_2_O_2_, and singlet oxygen (Mao Min et al., 2014). In addition, there are many enzyme systems in the organism that can convert O_2_ into O_2_-, such as the redox protein p66SHc between the inner and outer membranes of mitochondria, which uses the reduction amount of respiratory chain and oxidizes cytochrome C to produce H_2_O_2_, which is a new way to generate ROS in the mitochondrial respiratory chain (Giorgio et al., 2005).

Both mitochondrial cytochrome P450 and NADPH oxidase play an important role in regulating ROS production. Nox1-5 and Duox1-2 of NADPH family on the outer membrane of mitochondria are involved in the regulation of ROS production. In addition, cyclooxygenase, xanthine oxidase and lipid oxidase also affect ROS production in varying degrees (Mao Min et al., 2014). Many studies have found that many signal transduction pathways such as nuclear factors-κB (NF-κB), MAPK, phosphatidylinositol 3-kinase and p53 are all involved in ROS production. For example, tumor necrosis factor can activate apoptosis signal regulated kinase, increase ROS production, and up regulate the downstream influence factor c-Jun amino terminal kinase, thus activating the p38-MAPK pathway to induce cell contraction and apoptosis (Bigarella et al., 2014).

Mitochondria, as the primary consumption area of oxygen in cells, have always been regarded as oxygen sensing sites. They are released into the cytoplasm in the form of O_2_- and H_2_O_2_ signals, triggering downstream transcriptional signaling factors, such as hypoxia inducible factors, NF-κB and signal transducer and activator of transcription, thereby inducing pulmonary vascular inflammation, endothelial and smooth muscle contraction, and vasoconstriction and remodeling (Frazziano et al., 2014).

The ubiquity of ROS determines the universality of its damage to body functions, and it can be used as a signal molecule to affect downstream ion channels, such as the following oxygen sensitive voltage gated potassium channels, which increase potassium ions in the cytoplasm, depolarize pulmonary artery smooth muscle cells, thus activating voltage gated Ca^2+^ channels, and Ca^2+^ influx leads to hypoxic pulmonary vasoconstriction (Trebak et al., 2010).

Hypoxic PAH is a common systemic inflammatory syndrome, including scleroderma, systemic lupus erythematosus and pulmonary vascular disease (Balabanian et al., 2002). ROS plays an important role in the pathological changes of vascular diseases and is an important agonist of inflammatory response (Irani, 2000; Lennon and Salgia, 2014). ROS may promote the contraction and proliferation of PASMCs by increasing the secretion of proinflammatory chaperone cyclosporine binding protein A (Satoh et al., 2008). The increase of ROS production can promote the adhesion and migration of dendritic cells, and pretreatment with antioxidants can significantly inhibit this pathological phenomenon (Zhu et al., 2009).

Autophagy is a cellular process that relies on lysosomes to remove damaged organelles, maintain the stability of the intracellular environment, and enhance the tolerance of cells to starvation or hypoxia. ROS can induce autophagy, among which H_2_O_2_ and O_2_- are the most important inducers. The occurrence of mitochondrial autophagy can be reduced by reducing the content of O_2_- in mitochondria, which may be related to the ion release and lipid peroxidation induced by aconitase (Bensaad et al., 2009; Chen et al., 2009).

When mitochondria are damaged, toxic substances can be gathered into one mitochondria and then autophagic degradation can be induced through division (Aggarwal et al., 2013). Mitochondrial respiratory chain complexes I and II can regulate autophagic cell apoptosis through mitochondrial ROS, which further confirms the close relationship between mitochondrial ROS and autophagy (Chen et al., 2007). In addition, autophagy damage can negatively feedback to increase oxygen pressure, promote the accumulation of ubiquitinated proteins, ROS production and mitochondrial damage.

In the PAH rat model, inhibition of mitochondrial fusion protein DRP1/NADPH oxidase (NOX) pathway and autophagy related protein (Atg) - 5/Atg-7/Declin-1/microtubule related protein light chain 3 (LC3) dependent autophagy pathway in PASMCs can reduce PASMCs proliferation (Wu et al., 2019). ROS scavenger N-acetylcysteine can inhibit the formation of autophagy, and autophagy inhibitor chloroquine or 3-MA can also reduce the production of ROS, indicating that autophagy and the production of ROS have a close regulatory and feedback regulatory relationship, but the specific mechanism needs further study (Teng et al., 2012).

#### Signaling pathways in pulmonary vasoconstriction

Hypoxia can directly or indirectly promote the secretion of regulatory factors to regulate the blocking and activation of various kinase activities and signal pathways, thereby aggravating the contraction and remodeling of pulmonary arteries. Hypoxia can increase the calcium sensitivity of smooth muscle cells and regulate the intracellular Ca^2+^ concentration by activating Rho/Rho kinase signal, thereby promoting pulmonary vasoconstriction (Takemoto et al., 2002; Absi et al., 2019).

Mitochondrial aldehyde dehydrogenase 2 (ALDH2) is expressed in many tissues and organs *in vivo*, and its biological activity is closely related to a variety of cell functions, including proliferation and oxidative stress reaction (Matsumoto, 2019). It is found that activation of ALDH2 can reduce IL-1β, IL-6 and inflammatory protein one levels in serum of PAH mice, thereby reducing the process of PAH (Zhao et al., 2019). Calcineurin (CaN) is a serine/threonine protein phosphatase that depends on calcium and calmodulin. When the level of calcium ions in the cytoplasm increases, calcium ions can activate CaN by binding regulatory subunits and activating calmodulin binding. CaN activates the transcription factor T cell nuclear factor of activated T cells (NFATc) through dephosphorylation, and the activated dephosphorylated NFATc is then transported to the nucleus to further exert its function (Roy and Cyert, 2020). NFAT is a transcription factor family, including five members: NFATc1, NFATc2, NFATc3, NFATc4, and NFAT5. In recent years, some types of NFAT have been found to play an important role in the pathological process of hypoxic PAH. Jiang et al. (2018) found that NFATc3 was significantly increased in PASMCs under the hypoxia conditions. It has been found that mitochondrial ALDH2 and CaN can directly bind and regulate each other, and CaN also has a direct binding relationship with NFATc3 (Chen, 2021). Therefore, mitochondrial ALDH2 can inhibit the proliferation and contraction of pulmonary vascular smooth muscle cells by mediating the expression of CaN/NFATc3, thereby improving the contraction of pulmonary vessels in the acute phase and remodeling in the chronic phase (Chen, 2021).

### Mitochondrial glucose oxidation and pulmonary vasoconstriction

It is well known that pulmonary vascular complications are also high in patients with diabetes. This is mainly because glucose is the main energy source for maintaining vascular contraction. The main mode of glucose metabolism in blood vessels is glucose anaerobic fermentation. The stimulation of long-term metabolic disorder leads to the damage and increase of mitochondrial division in vascular endothelial and smooth muscle cells, which further affects the contraction and relaxation functions of blood vessels (Rajendran et al., 2013). With the deepening of research, there is also more and more evidence that mitochondria play an important role in the changes of vascular function caused by glucose metabolism.

Mitochondrial transport of molecules and ions is mainly mediated by specific transporters on the inner membrane surface. Among them, the impermeability of the inner membrane to ions causes the formation of ionic gradients on both sides of the mitochondrial inner membrane, which eventually results in an external positive and internal negative potential difference due to the difference in ion charge, which we call the mitochondrial membrane potential (ΔΨm) (Kauppinen, 1983). The electron transport chain pumps protons through the inner mitochondrial membrane to the mitochondrial membrane gap generating the proton gradient and ΔΨm. The proton gradient and ΔΨm are necessary for the synthesis of ATP. The opening of the uncoupling protein can reduce ΔΨm (Serviddio and Sastre, 2010). The maintenance of ΔΨm is essential for ATP synthesis.

The current consensus is that when metabolic demand and glucose energy supply increase, Ca^2+^ enters mitochondria to promote ATP production (Savignan et al., 2004). ΔΨm is also involved in maintaining the steady state of ROS. In the basic respiratory state of mitochondria, 2%–4% electrons leak out of the respiratory chain and combine with O_2_ to produce superoxide anion (Heinen et al., 2007). The number of leaked electrons from the respiratory chain increases as ΔΨm increases. In the resting state, the reduction of ΔΨm by 15% can reduce the ROS production by about 10 times (Gong et al., 2014). It has been found that the increase of ΔΨm in vascular smooth muscle cells can cause pulmonary vasoconstriction, while the decrease of ΔΨm can cause pulmonary vasodilation (Jie, 2017). It was also found that glucose oxidation could increase ΔΨm in vascular smooth muscle cells (Jie, 2017). When the expression of ΔΨm was inhibited, the effect of glucose oxidation on vascular contraction disappeared. This also shows that glucose oxidation causes vasoconstriction by increasing ΔΨm expression.

## Mitochondria-targeted treatment for PAH

The current treatment of PAH is mainly to dilate the pulmonary blood vessels, including stimulating the nitric oxide-cyclic guanosine monophosphate biological pathway, increasing prostacyclin effector receptors, and antagonizing the endothelin pathway (Humbert et al., 2014; Humbert et al., 2022). Although these drugs have certain effects on improving the life treatment, exercise capacity, and pulmonary artery pressure of PAH patients, they cannot reverse pulmonary vascular remodeling and improve their long-term prognosis (Humbert et al., 2010). This may be related to the unclear mechanism of PAH. Therefore, it is very necessary to further clarify the mechanism of PAH and find new therapeutic target strategies. In recent years, based on the basis of PAH genetics, cytology, and molecular biology, there are still some other potential therapeutic targets for PAH, including serotonin, vasoactive peptides, oxidative stress, tyrosine kinases, anti-inflammatory drugs, gene therapy and mitochondria-targeted therapy (Zolty, 2020). Unfortunately, although none of these approaches are currently FDA-approved for the treatment of PAH, we believe that mitochondrial-targeted therapy is a very promising treatment.

Promoting glucose oxidation, restoring mitochondrial imbalance, inhibiting mitochondrial fission and autophagy, and restoring mitochondrial calcium regulation are all promising experimental therapeutic strategies. Among them, targeting glucose metabolism may be an effective therapeutic strategy. *In vitro* experiments show that dichloroacetate (DCA) inhibitors can reverse PAH and Warburg effect in mice (Michelakis et al., 2002; Guignabert et al., 2009; Li et al., 2018; Li et al., 2020). Another study also demonstrated that combination of dichloroacetate and atorvastatin can regulate excessive proliferation and oxidative stress in PAH development *via* p38 Signaling (Li et al., 2020). The first-in-human clinical trial of a mitochondrial-targeted drug using DCA, an inhibitor of the mitochondrial enzyme pyruvate dehydrogenase, showed that it could improve hemodynamics in patients with genetic susceptibility to PAH, confirming that pyruvate dehydrogenase kinase mitochondrial the possibility of a target (Michelakis et al., 2017). The HK-2-restricted inhibitor 3--bromopyruvate (3-BrPA) was shown to reverse hypoxia-induced pulmonary vascular remodeling in a rat model (Chen F. et al., 2018). In addition, animal experiments showed that both 3PO targeting PFKFB3 and enolase one inhibitor AP-III-a4 could inhibit the formation of PAH by inhibiting glycolysis (Dai et al., 2018; Cao et al., 2019; Wang L. et al., 2021).

Strategies targeting the substrate switching theory of PAH metabolism are also a possible therapeutic approach. Studies have demonstrated that fatty acid oxidation (FAO), Ranolazine, and trimetazidine improve cardiac index and treadmill walking distance in rats while reducing lactate levels (Archer et al., 2013; Ma et al., 2020). A prospective study not only validated the safety of Ranolazine, but also showed improvement in WHO grade and right ventricular function, but unfortunately no change in hemodynamic parameters (Khan et al., 2015). In another multicenter study (NCT01839110), ranolazine improved right ventricular function, left ventricular end-diastolic volume, and biventricular stroke volume (Han et al., 2021). Although there are phase II studies of trimetazidine in PAH, no data have been published (Velayati et al., 2016). Antioxidants are another potential therapeutic approach targeting the disruption of redox homeostasis in PAH patients. Antioxidant system agonists, inhibitors of ROS production, and modulators of ROS-induced toxicity can reverse pulmonary artery remodeling and improve right heart function in animal models of PAH, such as sod-mimicking metalloporphyrin manganese (III) tetrakis (4-benzoate acid) porphyrin phenoline (MnTBAP), SOD-mimicking drug TEMPOL, MAOA inhibitor Clorgyline, GKT137831 inhibitor NOX4, etc., (Bertero et al., 2016; Dumas et al., 2018; Ge et al., 2018; Liang et al., 2022). Coenzyme Q (CoQ), an essential substance for mitochondrial and antioxidant responses, can also improve cardiac function in patients with PAH by improving mitochondrial and redox metabolism (Egnatchik et al., 2017).

In addition, therapeutic strategies targeting the inhibition of mitochondrial fission have also shown promising therapeutic prospects in animal models, such as dynamin-related protein 1, mitochondrial fission mediators (MiD49M, MiD51), up-regulation of MFN2, etc (Marsboom et al., 2012; Chen K. H. et al., 2018; Wu et al., 2019; Umezu et al., 2020; Feng et al., 2021; Xiao et al., 2022). Looking forward to the translation of these drugs from the laboratory to the clinic.

Despite the growing number of treatments for PAH, the traditional single treatment regimen cannot significantly improve the survival rate of patients with PAH, nor can it significantly reduce the morbidity and mortality. Current treatment methods recommend one or more combination therapy of different biological pathways (Pulido et al., 2013; Galiè et al., 2015; Sitbon et al., 2015; Lajoie et al., 2016; Galiè et al., 2019; White et al., 2020). With combined therapy, the 5-year survival rate for PAH has improved by nearly 30% compared to 34% in 1991, which has been confirmed by many large clinical trials (Pulido et al., 2013; Galiè et al., 2015; Sitbon et al., 2015; Ruopp and Cockrill, 2022). However, studies have shown that adding selexipag to macitentan and tadalafil does not improve pulmonary vascular resistance in patients with PAH (Burki, 2020). Therefore, more and larger randomized clinical studies are needed to tell patients which drug combinations are effective and which drug combinations are ineffective.

Mitochondrial dysfunction is a major cause of pulmonary arterial hypertension, so mitochondrial transplantation may be another potential therapy. Although mitochondrial transplantation currently exists only in laboratory models of PAH and can improve pulmonary vasoconstriction, right heart function, and pulmonary vascular remodeling, it is still far from clinical application (Maarman, 2022). We believe that researchers will overcome various difficulties to further prove the possibility of mitochondrial transplantation.

## Conclusion

PAH remains a global health problem, that is, not yet fully recognized and understood. About 80% pulmonary hypertension patients are in developing countries. Although heart and lung diseases are the main underlying diseases in patients with pulmonary hypertension, HIV, schistosomiasis or rheumatic heart disease also play a role (Hoeper et al., 2016). But puzzlingly, disease progression and death in these patients were associated with pulmonary hypertension, but not with the underlying disease (Hoeper et al., 2016). The causes and mechanisms of death in patients with PAH remain a mystery. Through in-depth dissection of the relationship between mitochondrial metabolism and PAH, it was found that mitochondrial dysfunction is the main driver of PAH. Mitochondrial dysfunction can affect pulmonary vasoconstriction and remodeling in many ways. Pulmonary vasoconstriction and remodeling are two different stages of the formation of PAH. The development of the two stages is independent, interactive and interactive. This study combed the relationship between mitochondria and pulmonary vasoconstriction and remodeling. However, the synergistic or antagonistic mechanism between mitochondrial metabolic pathways is still unclear, and more research is needed to further clarify. We expect to find more mechanisms to help us find targeted drugs to accurately control PAH. In addition, in clinical practice, adherence to drug therapy may be one of the key factors for successful treatment of PAH patients.

There are some flaws in this study. First of all, the level of evidence in this paper is mainly based on the PAH-related studies that have been published so far, so the quality is also limited. At the same time, although a large number of relevant literature have been fully incorporated in the process of writing this article, it is inevitable that some important literature information may be omitted. Additionally, this study did not conduct a formal quality assessment of each article included. Finally, we only discussed the relationship between some mitochondrial dysfunction and PAH. However, the research contents of endothelial cell or fibroblast mitochondrial damage and mitochondrial mechanical mechanics in PAH were not summarized and discussed. At the same time, the function of each ion channel in mitochondria has not been discussed in depth. However, we will conduct an in-depth summary and discussion in this regard.
